# The effects of transcranial alternating current stimulation (tACS) at individual alpha peak frequency (iAPF) on motor cortex excitability in young and elderly adults

**DOI:** 10.1007/s00221-018-5314-3

**Published:** 2018-06-26

**Authors:** Shane Fresnoza, Monica Christova, Theresa Feil, Eugen Gallasch, Christof Körner, Ulrike Zimmer, Anja Ischebeck

**Affiliations:** 1 Institute of Psychology, University of Graz, Graz, Austria; 2 Otto Loewi Research Center, Physiology Section, Medical University of Graz, Graz, Austria; 3 Institute of Physiology, Medical University of Graz, Graz, Austria; 4 BioTechMed, Graz, Austria; 5 Department of Physiotherapy, University of Applied Sciences FH-Joanneum Graz, Graz, Austria; 6 Faculty of Human Sciences, Medical School Hamburg (MSH), Hamburg, Germany

**Keywords:** Alpha oscillation, Aging, Electroencephalogram, Transcranial alternating current stimulation, Transcranial magnetic stimulation, Neuronal entrainment

## Abstract

Transcranial alternating current stimulation (tACS) can modulate brain oscillations, cortical excitability and behaviour. In aging, the decrease in EEG alpha activity (8–12 Hz) in the parieto-occipital and mu rhythm in the motor cortex are correlated with the decline in cognitive and motor functions, respectively. Increasing alpha activity using tACS might therefore improve cognitive and motor function in the elderly. The present study explored the influence of tACS on cortical excitability in young and old healthy adults. We applied tACS at individual alpha peak frequency for 10 min (1.5 mA) to the left motor cortex. Transcranial magnetic stimulation was used to assess the changes in cortical excitability as measured by motor-evoked potentials at rest, before and after stimulation. TACS increased cortical excitability in both groups. However, our results also suggest that the mechanism behind the effects was different, as we observed an increase and decrease in intracortical inhibition in the old group and young group, respectively. Our results indicate that both groups profited similarly from the stimulation. There was no indication that tACS was more effective in conditions of low alpha power, that is, in the elderly.

**Electronic supplementary material:**

The online version of this article (10.1007/s00221-018-5314-3) contains supplementary material, which is available to authorized users.

## Introduction

In the present study, we investigated the effects of transcranial alternating current stimulation (tACS) at the individual alpha peak frequency (iAPF) on corticospinal excitability and intracortical inhibition and facilitation in old and young healthy individuals. TACS can influence the oscillatory activity of the brain. We expected different effects of the stimulation between old and young participants because oscillatory activity (e.g. alpha frequencies) undergoes changes due to aging. In the sensorimotor cortex, the 8–13 Hz oscillation called mu or Rolandic rhythm attenuates (event-related desynchronization or ERD) during the induction of actual movements, motor imagery and the processing of body-related action verbs (Pfurtscheller and Neuper 1993, 1994, 1997; Niccolai et al. 2014). On the other hand, large and more synchronized mu rhythms (event-related synchronization or ERS) are prominent after movement and during reading (Pfurtscheller and Neuper 1993; Pfurtscheller and Lopes da Silva 1999). At the parieto-occipital area, the mu rhythm overlaps with a 10 Hz oscillation called the “classical” alpha rhythm thought to primarily reflect visual processing in occipital networks (Pineda 2005). However, ERD of the alpha rhythm was also evident during movement preparations and iconic gesture observation (Deiber et al. 2012; Quandt et al. 2012). Furthermore, ERD in the alpha band may not occur in isolation but can be accompanied by ERS in neighbouring cortical areas that correspond to the same or to another modality of information processing (Pfurtscheller 2003). For instance, during repetitive brief finger movements and during the preparation of self-paced fist closing, central mu rhythm undergoes desynchronization while the alpha rhythms undergoes synchronization (Westphal et al. 1993; Gerloff et al. 1998; Pfurtscheller et al. 2000). Although the exact physiological role of the cortical 8–13 Hz oscillation in the neural control of movement is not yet completely understood, magneto- and electroencephalographic (EEG/MEG) studies strongly suggest that the decreased oscillatory alpha activity facilitates processing in a given region, whereas increased alpha activity serves to actively suppress irrelevant or interfering processes (Haegens et al. 2011).

In aging, the posterior alpha frequency slows down and its peak power and coherence decrease (Giaquinto and Nolfe 1986; Böttger et al. 2002; Richard Clark et al. 2004; Grandy et al. 2013; Vysata et al. 2014). The decreased magnitude of the alpha rhythm correlates with global cognitive decline during physiological aging (Babiloni et al. 2006; Ishii et al. 2017). Additionally, there is an anterior shift of ongoing alpha activity over the fronto-central areas in healthy elderly as well (Yordanova et al. 1998; Kolev et al. 2002). Similarly, widespread suppression of the mu rhythm over the motor and premotor areas is a common finding in elderly subjects during finger movements compared with young subjects (Derambure et al. 1993; Mary et al. 2015). This widespread spatial distribution and more uniform flat curve of power decrease in the elderly compared to young participants was also observed during cued finger movements, pinches and a whole hand grip task in sensorimotor areas and was linked to age-related changes in the neural coding of skilled motor behaviour (Quandt et al. 2016). On the other hand, some elderly individuals failed to show a significant mu power increase at the sensorimotor cortices during inhibition of learned movements. This finding suggests a deficit in the generation and enhancement of local inhibitory mechanisms at the sensorimotor cortices in aged brains (Bönstrup et al. 2015). Cortical disinhibition secondary to a decline in mu and alpha activity has been linked to the increasing incidence of epilepsy in several neurological disorders of the elderly such as Alzheimer’s disease (Hitomi et al. 2011; Neto et al. 2015). Interestingly, decreased mu-related inhibition of the visual cortex and alpha-related inhibition at the sensory-motor networks at rest were also the prominent findings in photosensitive epileptic young patients (Vaudano et al. 2017). Additionally, cortical hyper-excitability due to disinhibition is proposed to serve as an early compensatory mechanism to execute voluntary movements in physiological aging. When this compensation fails with aging, impaired motor control during contractions, a profound decrease in postural stability and overall low manual motor performance become more evident (Heise et al. 2013; Papegaaij et al. 2014; Opie and Semmler 2015). Therefore, interventions that can modulate the mu and alpha oscillation to enhance its role in cortical inhibition would be useful as an adjunct for motor rehabilitation and treatment of disorders associated with cortical hyper-excitability in the elderly.

TACS offers the possibility to directly modulate cortical oscillations such as alpha (Antal and Paulus 2013). The exact neurophysiological mechanism underlying tACS is unknown, but the effects during stimulation were mainly attributed to neuronal entrainment or the frequency and phase alignment of endogenous oscillatory activity to the applied periodic current (Thut et al. 2011; Helfrich et al. 2014). In contrast, the origin of the stimulation after-effects is comparatively unclear; they could be due to entrainment echoes and spike-timing-dependent plasticity (Veniero et al. 2015). TACS-induced effects seem to depend on the frequency, intensity and the phase of the stimulation (Antal and Paulus 2013). Stimulation at iAPF seems most promising, since amplifying network activity is more robust when the stimulation is at the area’s resonance or “natural” frequency (Battleday et al. 2014). TACS applied at fixed alpha frequencies (e.g.10 Hz) or at iAPF increased the posterior EEG alpha power after stimulation (Zaehle et al. 2010; Neuling et al. 2012a; Helfrich et al. 2014; Kasten et al. 2016). Additional studies showed that the effect of stimulation was state-dependent. Post stimulation posterior alpha power and phase coherence were significantly increased only under conditions of low endogenous prestimulation alpha power and could not be further enhanced under conditions of high alpha power (Neuling et al. 2013; Ruhnau et al. 2016). The concurrent brain state (e.g. resting vs during task performance) may also change the susceptibility of a cortical area to the effect of stimulation. In the motor cortex, alpha tACS increased the MEP size only during motor imagery and had no effect without it (Feurra et al. 2013). On the other hand, enhanced performance in a mental rotation task is associated with enhanced reference alpha power, which was not evident during the resting periods (Kasten and Herrmann 2017). At present, there is no available evidence that the state-dependent effect of tACS stimulation (e.g. during eyes open or eyes closed) in the alpha frequency range may extend to conditions (e.g. young and aged brain) characterized by decreased alpha power and slowing of iAPF such as the aged brain. Investigating the effects of tACS on the mu alpha oscillation in healthy aging can offer insights into age-related changes in cortical excitability and can serve as an additional treatment option of illnesses common to the elderly.

Cortical excitability as well as inhibition can be measured using transcranial magnetic stimulation (TMS). TMS induces an electric current in the brain by application of a transient magnetic field. The induced current can depolarize neuronal cell membranes and give rise to an action potential. When the hand area of the motor cortex is targeted, a finger movement or motor-evoked potential (MEP) can be elicited (Rossini and Rossi 2007). It is a safe and valuable tool for studying the changes in the aged motor cortex. In the present study, we used TMS to explore the effects of tACS on motor cortical excitability. Due to the strong and reciprocal connection between the parietal and motor areas, and because of their distinct but overlapping oscillation during motor behavior we hypothesized that stimulation of the motor cortex using the individual alpha peak frequency (iAPF) from the parietal areas could modulate motor cortical excitability. We also hypothesized that the effect of tACS at iAPF would be greater in scenarios with reduced alpha power and slower oscillations, as typically present in the elderly. We measured corticospinal excitability using single-pulse TMS and the recruitment curve, as well as intracortical inhibition (SICI) and facilitation (ICF) using a paired-pulse TMS paradigm. We expected a more robust increase in corticospinal excitability in the old group after tACS stimulation. On the other hand, we expected intracortical inhibition and facilitation to be modulated differently by tACS in both groups. This is the first electrophysiological study that tested the effects of tACS at iAPF on older adults, and therefore contributes to the investigation of the mechanism of action of tACS on the aged motor cortex.

## Materials and methods

### Participants

Twelve healthy young adults (6 males) with a mean age of 24.16 years (range 18–28, SD 3.12) and 12 healthy elderly adults (4 males) with a mean age of 61.16 years (range 56–67, SD 3.48) were recruited for the study. The young participants were all college students, while the group of older participants composed of ten retirees and two participants who were still working. The older group had 14.33 ± 0.94 mean years of education. All participants were right handed according to the Edinburgh Handedness Inventory (Oldfield 1971). All were screened for possible neuro-psychiatric disorders and contraindications to TMS and tACS (Poreisz et al. 2007; Rossi et al. 2009). Participants who were taking medications during or up to 2 weeks before the study and those with maintenance drugs were also excluded. They gave written informed consent prior to the experiment. The study conformed to the Declaration of Helsinki and was approved by the Ethics Committee of the Medical University Graz.

### TACS stimulation

TACS was applied through a pair of saline-soaked surface sponge electrodes connected to a battery-driven stimulator (ELDITH DC-stimulator, NeuroConn, Germany). A 35 cm^2^-electrode was positioned over the left motor cortex’s representational area of the right FDI, which was identified using single pulse TMS (see Experimental Procedure). The second electrode measuring 100 cm^2^ was attached to the right supraorbital area. Modelling studies suggest that adequate current can reach the motor cortex with this electrode montage without the influence of head fat distribution that may be different between young and old people (Miranda et al. 2006; Truong et al. 2013). Stimulation focality was ensured by the electrode size differences, since the current density was higher over the motor cortex (0.043 mA/cm^2^) than at the supraorbital area (0.015 mA/cm^2^) (Nitsche et al. 2007; Faria et al. 2011). We delivered a 1.5 mA peak-to-peak current (no DC offset, no phase shift), because lower intensities (0.4–1.0 mA) did not modify EEG power and MEP amplitudes in the motor cortex (Antal et al. 2008; Wach et al. 2013). On the other hand, intensities of 1.0-1.5 mA increased MEP amplitudes and BOLD responses (Groppa et al. 2010; Moliadze et al. 2012; Cabral-Calderin et al. 2015). Regarding the stimulation duration, 5 min of stimulation has no effect, while 20 min decrease MEP amplitudes and had no impact on intracortical inhibition (Antal et al. 2008; Zaghi et al. 2010). In another study, 10 Hz tACS for 10 min (1 mA intensity) had no effect on MEP amplitudes but was able to shorten cortical silent period indicating interference with inhibitory pathways (Wach et al. 2013). Therefore, since we are using a higher current intensity (1.5 mA) than previous studies, we believe that a fixed stimulation duration of 10 min would be enough to modulate cortical excitability. To minimize the tingling skin sensation, the impedance during stimulation was maintained below 10 kΩ. The current was slowly ramped-up and down for 10 s at the start and end of stimulation, respectively. To ensure that participants experienced a similar skin sensation in the sham condition as in the real stimulation condition, the current during sham stimulation was applied for only 30 s (with 10 s current ramping) and then turned off automatically.

### EEG recording

Resting state EEG (10–20 system) was recorded over five scalp locations (F3, F4, Cz, Pz and Oz) with Brain Vision Recorder (Brain Products GmbH, Munich, Germany). The ground electrode was located on the forehead (FPz electrode), while the reference electrode was on the left mastoid. To detect eye movements, an electrooculogram (EOG) was recorded using lateral and glabellar electrodes. EEG and EOG signals were digitized with a sampling rate of 500 Hz and band pass filtered ranging from 0.1 to 100 Hz. An additional 50 Hz notch filter was applied. Electrode impedances were kept below 5 kΩ for the EEG recordings and below 10 kΩ for the EOG recording.

### TMS measurements

Changes in cortical excitability were measured using two monophasic Magstim 200 magnetic stimulators connected via a Bistim module (The Magstim Company, Whitland, Dyfed, UK). A figure-of-eight coil (9 cm outer loop diameter) was used to deliver the pulses. The coil was positioned on the scalp over the left motor cortex at the optimal site for stimulating the contralateral right first dorsal interosseous (FDI) muscle. The intersection of the coil was kept tangential to the scalp, the handle pointing backward and laterally at a 45° angle from the midline sagittal axis. This position induces a posterior–anterior current flow which is efficient in activating the corticospinal system trans-synaptically (Di Lazzaro et al. 1998). Surface electromyography (EMG) recordings were obtained from the FDI muscle through a pair of Ag–AgCl surface electrodes in a belly-tendon montage. EMG signals were amplified, band pass filtered (8–2000 Hz), digitized (sampling rate 10 kHz) and recorded using Micromed software (System plus software). To ensure the absence of any voluntary muscle activity before the MEP, EMG signals were displayed and assessed for contraction artifacts during TMS measurements.

Two TMS paradigms were used to monitor the changes in corticospinal excitability: single-pulse TMS with the intensity set at SI 1 mV (stable 1 mV) threshold and the input/output (*I*/*O* curve) or recruitment curve. The MEPs peak-to-peak amplitude elicited from the left motor cortex representation of the FDI muscle was used to assess excitability changes. Motor thresholds depend on the excitability of neural elements that elicit an action potential in response to a TMS pulse (Ziemann et al. 2015). In our study, the SI 1 mV threshold was defined as the minimum output of the TMS stimulator that elicits an average of 1 mV peak-to-peak amplitude from 25 recorded MEPs. On the other hand, the *I*/*O* curve reflects neuronal membrane excitability changes in response to increasing intensities set at the resting motor threshold (RMT) (Lazzaro et al. 2003). The slope of the recruitment curve is related to the strength of corticospinal projections and was steeper in muscles with a lower CMT or cortical motor threshold (Chen et al. 1998). The increasing intensities recruit neurons away from the point of stimulation, causing a linear increase in the MEP amplitudes (Chen 2000). RMT was defined as the minimum stimulator intensity that elicits a reliable MEP amplitude of about 50 µV in at least three out of six consecutive trials in a relaxed FDI muscle (Rossini et al. 2015). For the *I*/*O* curve, we recorded 15 MEPs for each intensity (110, 130 and 150% of RMT).

For monitoring intracortical excitability, we also used the peak-to-peak MEP amplitude induced in the left motor cortex by means of a paired-pulse TMS paradigm called short-interval intracortical inhibition (SICI) and intracortical facilitation (ICF). In SICI/ICF paradigms, paired magnetic pulses were administered through the same stimulating coil at different inter-stimulus intervals (ISIs) (Kujirai et al. 1993). The first pulse or the conditioning stimulus (CS) was set at 80% of the active motor threshold (AMT), while the second pulse or test stimulus (TS) was set at the SI 1 mV threshold. AMT was defined as the minimum stimulator intensity that can evoke an MEP of about 50 µV in three out of six trials in a moderately active muscle (Awiszus 2003). The CS inhibits the MEP amplitude elicited by the TS for short intervals (1–5 ms), whereas it enlarges it at longer intervals (6–20 ms) (Kujirai et al. 1993; Ziemann et al. 2015). The GABA_A_-mediated inhibitory post-synaptic potentials (IPSPs) were suggested to be responsible for SICI (Ziemann et al. 1996a; Hanajima et al. 1998; Ilić et al. 2002). On the other hand, ICF likely reflects a combined *N*-methyl-d-aspartate (NMDA) and GABA_A_ receptor-mediated facilitation distinct from the SICI network (Hanajima et al. 1998; Ziemann et al. 2015). In the present study, we measured SICI at 2 ms ISI because inhibition was reported to be maximal and expressed without contamination by short-interval intracortical facilitation (SICF), ICF or any refractoriness of neural elements at this interval (Ziemann et al. 1996b; Di Lazzaro et al. 2000; Roshan et al. 2003; Peurala et al. 2008; Wagle-Shukla et al. 2009). For ICF, we chose an ISI of 13 ms because we expected a maximal increase in MEP amplitude at the median ISI (6–20 ms) known to induce MEP facilitation. We recorded 15 MEPs evoked by the TS and 15 MEPs evoked by the paired pulses (CS + TS) for SICI and ICF, separately.

### Experimental procedure

**Fig. 1:**
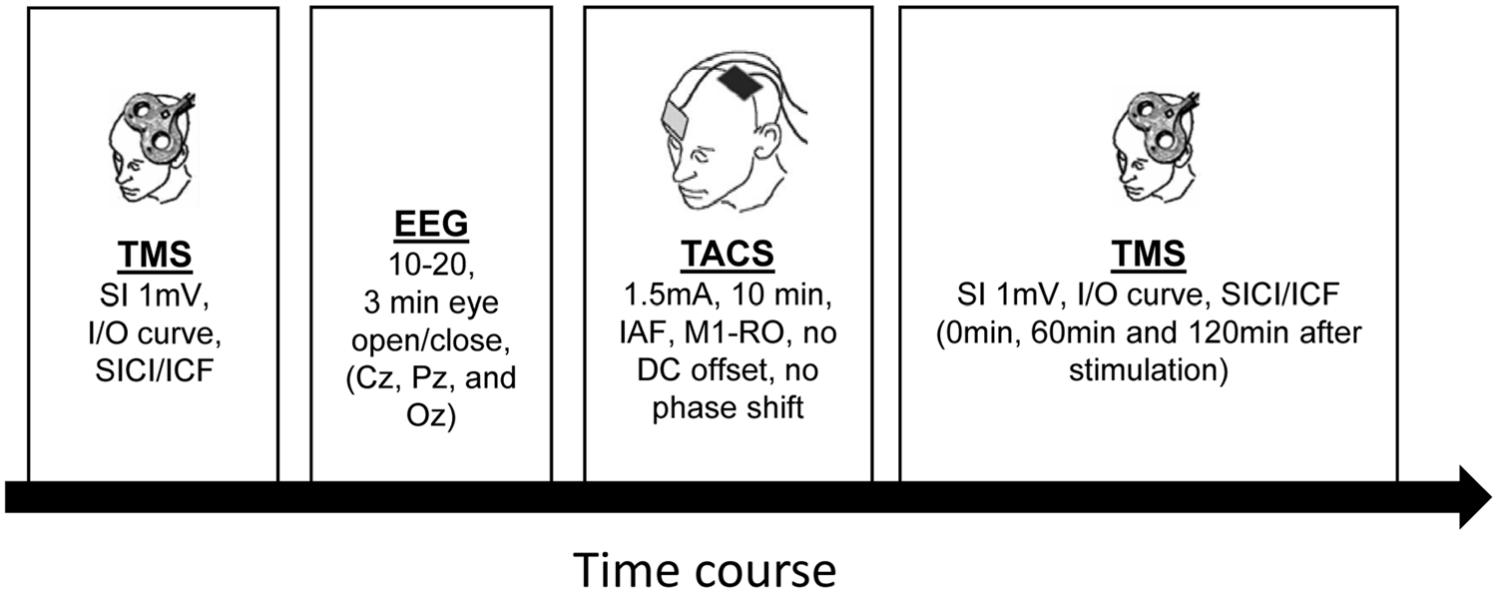
Course of the experiment. First, baseline TMS measures of corticospinal (single-pulse TMS and *I*/*O* curves) and intracortical excitability (SICI/ICF) were recorded. Immediately after, a 3-min, eyes-open and eyes-closed spontaneous EEG measurement was obtained. EEG data during the eyes-open condition was analyzed to identify the iAPF. Then tACS was administered for 10 min using the participant’s iAPF. This was followed by the TMS measurements. *SI 1 mV* stable 1 mV threshold for single-pulse TMS, *I*/*O* input/output, *SICI* short interval intracortical inhibition, *ICF* intracortical facilitation, *EEG* electroencephalogram, *iAPF* individual alpha peak frequency

The experiment was conducted in a single blinded, randomized, sham-controlled design. Each participant underwent two experimental sessions (real tACS and sham tACS) separated by an interval of at least 1 week to avoid carry-over effects. Due to the diurnal variations in the alpha activity, all measurements were carried out in the middle of the day (12:00–15:00) (Higuchi et al. 2001). The experiment started with the baseline TMS measurements (Fig. 1). Participants were seated in a comfortable reclining chair with head and arm supports. They were asked to relax and keep their eyes open during the measurements. EMG electrodes were attached to the right FDI using a belly-tendon montage. Then using TMS, the left motor cortex “hotspot” was identified and baseline corticospinal (SI 1 mV and *I*/*O* curves) and intracortical excitability (SICI/ICF) measurements were recorded (Fig. 1). EMG electrodes and motor cortex hotspot locations were marked with a permanent skin marker to ensure their constant positions throughout the experimental sessions. Afterwards, participants were transferred to an adjacent room that was sound proof and electrically shielded for the EEG recording. To have a stable electrode-scalp contact, the tACS electrodes were fixed underneath the EEG cap using rubber strips. Then EEG signals were recorded during a 3-min eyes open (with central fixation) and 3-min eyes closed period. The baseline TMS measurements, EEG preparation and recordings lasted for approximately 40 min. EEG data were immediately analyzed using a customized Matlab-based algorithm (Matlab R2016a, The MathWorks Inc., Natick, MA, USA). During this period, EEG electrodes were detached (cap was kept) and the participants were transferred back to the TMS room. The tACS stimulation immediately started after the identification of the participant’s iAPF. During a short pause (maximum 3 min), the EEG cap and tACS electrodes were removed. Post-tACS stimulation EEG measurements were not conducted, to assess the immediate impact of the stimulation on cortical excitability with TMS. Additionally, it would be difficult to dissociate the possible impact of the baseline TMS measurements from the effect of tACS stimulation on the EEG signal in the motor area (Thut et al. 2003; Fuggetta et al. 2005; Stamoulis et al. 2011; Manganotti et al. 2012; Mutanen et al. 2013). The TMS protocols were repeated immediately, 60 and 120 min after stimulation. One experimental session including the preparations lasted for about 3 h.

## Data analysis and statistics

### EEG analysis

Data were preprocessed and analyzed using Brain Vision Analyzer software (BrainProducts GmbH) and Matlab R2015a (The MathWorks Inc., Natick, MA, USA). The resting-state, eyes-open EEG data were first epoched into 4000 ms segments. Then artifacts like eye blinks, electrocardiographic and other non-cerebral signals were removed. A fast Fourier transformation (FFT) algorithm with a 0.25 Hz resolution and a 50% Hanning window was used to calculate the power spectral density with a confidence interval boundary of 90% (Welch’s method). The iAPF was defined as the frequency where the maximum power occurs within the alpha range of 8–13 Hz (Klimesch 1999). We determined the iAPF from the Pz electrode because contamination from the baseline TMS measurements in the left motor cortex is less likely for EEG signals recorded from the parieto-occipital areas.

### MEP analysis

Statistical analyses were conducted using SPSS software (SPSS 24, IBM Corp., Armonk, NY, USA). The peak-to-peak MEP amplitudes (µV) evoked by single pulse TMS (SI 1 mV), by each RMT intensity (110, 130 and 150%) in the recruitment curve and by paired-pulse TMS paradigm (SICI/ICF) before and after tACS stimulation were modelled separately using linear mixed-effect models. Shapiro–Wilk test and Levene’s test was used to assess normal data distribution and homogeneity of variances, respectively. Our dependent variable includes all raw MEPs for the single-pulse TMS and recruitment curve and normalized (to the respective TS) MEPs for SICI/ICF. We excluded MEPs that were two standard deviations away from the participant’s mean and those evoked by a TMS pulse that was preceded by muscle contraction artifact. Each participant was specified as a random factor (random intercept) and the between-subjects factor group (young vs old), and the within-subjects factors stimulation (sham and tACS) and time (prestimulation, 0, 60 and 120 min after stimulation) were treated as fixed factors. RMT was included as the within-subject fixed factor “intensity” in the model for the recruitment curve. For the separate model of SICI and ICF, the fixed factor “ISI” corresponding to the 2 and 13 ms inter-pulse interval was excluded in the final model. To select a parsimonious model, we started with a minimal model and incrementally added the predictors (Barr et al. 2013). The baseline model only contained the random effects variable (intercept) to examine the individual variation in the dependent variable without regard to the other predictors (Singer and Willett 2003). We then added the within-subject factors followed by the between-subject factor including their respective interactions. By adding a factor to the model one-at-a-time, we were able to compare the Akaike Information Criterion (AIC) values that indicate model adequacy. This method can determine over fitting in the model because it penalizes the likelihood function for having too many parameters. A decrease or increase in AIC value (> 2) upon the addition of a factor indicates model fit improvement or worsening, respectively (Burnham and Anderson 2002). Maximum likelihood estimation (Compound Symmetry models) was used to estimate the parameters of the models. However, the AIC value only compares one model to the next and does not indicate the absolute fit of the model to the data, therefore we also calculated the Akaike weight of each model (Burnham and Anderson 2002). The Akaike weight compares all possible models and determines which model will come out best most of the time. Additionally, we excluded non-significant factors in the final models except when they were involved in significant higher interactions. The tolerance and variance inflation factor checked for possible collinearity in the final models. We also added the iAPF to each final model as a covariate to assess if the prestimulation alpha frequency had an impact on cortical excitability changes. We calculated Cohen’s *d* as a measure of effect size since SPSS does not provide effect size values for mixed models. Significant findings from the models were explored using Bonferroni post hoc tests (adjusted for multiple comparisons). For single comparisons (e.g. sham vs tACS time points), we performed paired sample *t* tests. A *p* value of < 0.05 was considered significant for all statistical analyses. All values were expressed as mean ± standard error of mean (SEM).

## Results

All participants tolerated the TMS measurements and tACS stimulation well. During active tACS stimulation, five participants (two young, three old) reported phosphene sensations but were able to finish the experiment. No other adverse effects like headache, dizziness, nausea and vomiting noted. Overall, old participants received stimulation at slower frequencies since their pre-stimulation (average of sham and tACS session) iAPF and power (mean alpha frequency: 9.39 ± 0.27 Hz; mean alpha power: 10.56 ± 1.19 dB) was significantly slower and lower than the young group (mean alpha frequency: 10.29 ± 0.15 Hz; mean alpha power: 15.3 ± 2.73 dB). In the final model for single-pulse TMS, we did not include the between-subject factor “group” because it’s main effect [*F* (1, 24.01) = 1.55, *p* = 0.250] and three-way interaction effect [*F* (3, 4654.21) = 0.57, *p* = 0.633] were not significant. Additionally, both main effect and three-way interactions did not improve the model fit based on the AIC values (see Online Resource 1) and Akaike weights (see Online Resource 2). On the other hand, the factor group was included in the final model for the recruitment curve, SICI and ICF. All modelled data were normally distributed after log transformation (Shapiro–Wilk test) and the variances were equal for each group (Levene’s test) (all *p* > 0.05). Tolerance range and variance inflation factors were equal to 1.00 in all the final models indicating that multicollinearity had no effect on the significant findings revealed by the final models. The main effect of iAPF (as a covariate) was not significant for the single-pulse TMS [*F* (1, 4557.20) = 1.131, *p* = 0.288], recruitment curve [*F* (1, 7329.392) = 1.666, *p* = 0.164], SICI [*F* (1, 2264) = 3.249, *p* = 0.072] and ICF [*F* (1, 2344) = 2.712, *p* = 0.100] paradigms. Therefore, we excluded it in the final analysis. The results from the analysis of the final models were as follows.

### Corticospinal excitability

**Fig. 2:**
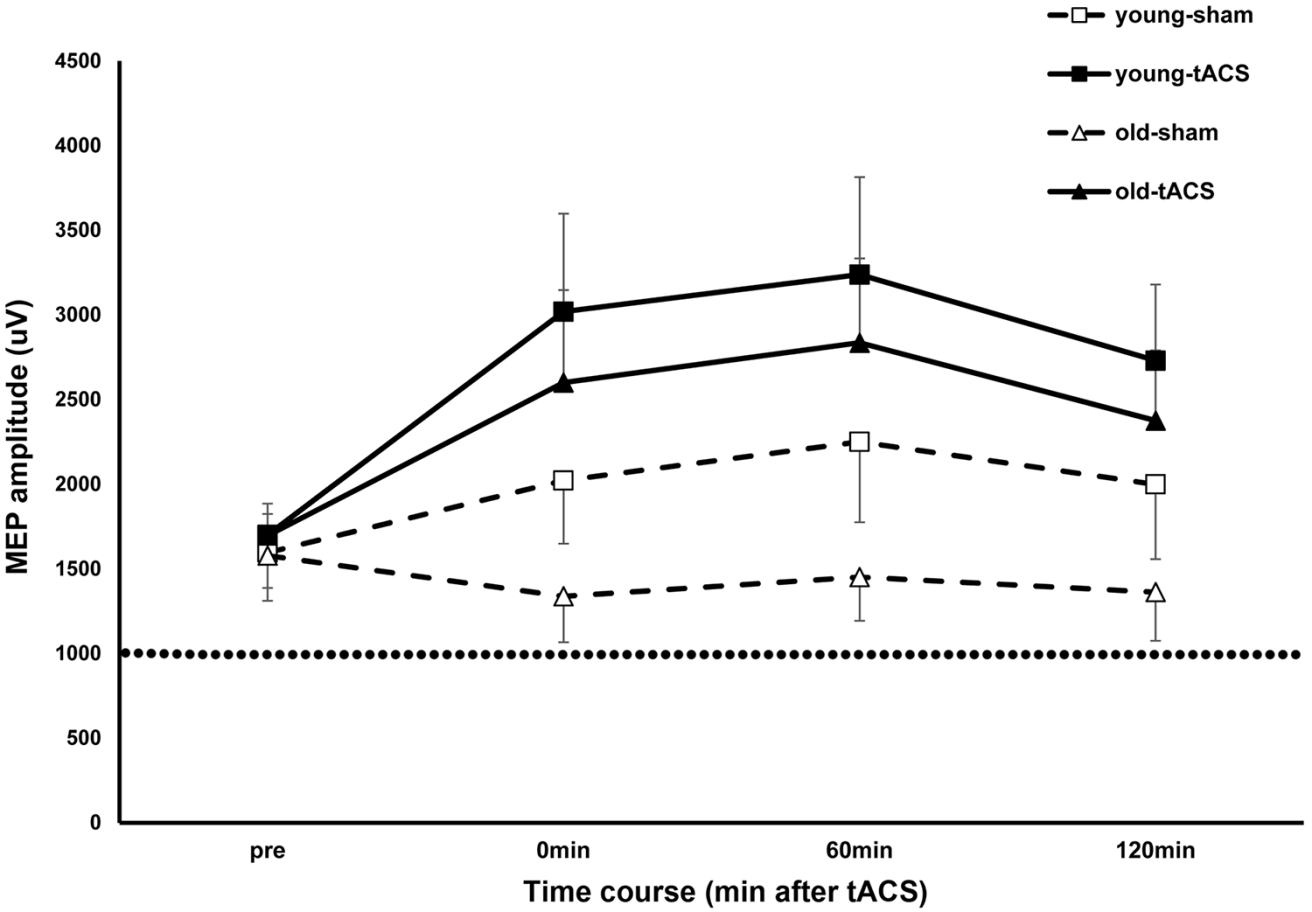
The effect of tACS at iAPF on corticospinal excitability measured by single pulse TMS. The *x*-axis displays the time points before and after stimulation (Pre = before stimulation). The *y*-axis displays the non-normalized MEP amplitudes (mean ± SEM as error bars) in µV. After tACS stimulation, corticospinal excitability increased in both groups. Filled symbols indicate MEP amplitudes in the tACS stimulation conditions

The results of the linear mixed model showed that there were significant changes in corticospinal excitability (MEP amplitudes) over time [significant main effect of time: *F* (3, 4654.21) = 60.50, *p* ≤ 0.001, *d* = 0.410] (Table 1). Bonferroni-corrected post hoc tests for the factor time showed significant differences between the MEP amplitudes immediately after, 60 min after, and 120 min after stimulation compared to baseline (all *p*s < 0.001). The results also showed that sham and tACS stimulation differentially modulated corticospinal excitability [significant main effect of stimulation: *F* (1, 4654.43) = 391.71, *p*  ≤ 0.001, *d* = 0.943]. MEP amplitudes increased by an average of 76.13% in the young group and 53.49% in the old group compared to baseline after real tACS (Fig. 2). On the other hand, MEP amplitudes only increased by 30.95% in the young group and 12.31% in the old group compared to baseline after sham stimulation [significant stimulation × time interactions: *F* (3, 4654.21) = 32.76, *p*  ≤ 0.001, *d* = 0.654].

**Table 1:** Results of the linear mixed model (LMM) performed for the single-pulse TMS, *I*/*O* curve and SICI/ICF measurements

	Numerator *df*	Denominator *df*	*F*-value	*p* value	Cohen’s *d*
SI 1 mV					
Stimulation	1	4654.43	391.71	< 0.001*	0.943
Time	3	4654.21	60.50	< 0.001*	0.410
Stimulation × time	3	4654.21	32.76	< 0.001*	0.654
*I*/*O* curve					
Group	1	24.01	0.47	0.501	0.197
Stimulation	1	7344.47	30.60	< 0.001*	0.190
Time	3	7344.18	19.81	< 0.001*	0.153
Intensity	2	7344.15	2159.34	< 0.001*	0.610
Group × stimulation	1	7344.47	50.33	< 0.001*	0.131
Group × time	3	7344.18	1.47	0.222	0.089
Group × intensity	2	7344.15	23.85	< 0.001*	0.305
Stimulation × time	3	7344.21	11.28	< 0.001*	0.140
Stimulation × intensity	2	7344.13	2.57	0.076	0.125
Time × intensity	6	7344.07	2.22	0.038*	0.106
Group × stimulation × time	3	7344.21	26.54	< 0.001*	0.093
Group × stimulation × intensity	2	7344.13	16.81	< 0.001*	0.048
Group × time × intensity	6	7344.07	0.65	0.693	0.057
Stimulation × time × intensity	6	7344.07	0.81	0.565	0.163
Group × stimulation × time × intensity	6	7344.07	5.16	< 0.001*	0.214
SICI					
Group	1	77.60	0.40	0.530	0.154
Stimulation	1	2437.23	7.24	0.007*	0.162
Time	3	2437.26	5.04	0.002*	0.164
Group × stimulation	1	2439.46	7.92	0.005*	0.259
Group × time	3	2437.25	17.19	< 0.001*	0.196
Stimulation × time	3	2437.50	15.80	< 0.001*	0.237
Group × stimulation × time	3	2437.20	6.33	< 0.001*	0.678
ICF					
Group	1	23.99	6.56	0.017*	0.744
Stimulation	1	2512.15	11.80	0.001*	0.215
Time	3	2511.52	5.61	0.001*	0.145
Group × stimulation	1	2512.15	3.69	0.055	0.170
Group × time	3	2511.51	5.60	0.001*	0.278
Stimulation × time	3	2511.59	1.24	0.293	0.198
Group × stimulation × time	3	2515.59	0.60	0.617	0.178

For the LMM (random intercept model), each participant was treated as a random factor. The between-subjects factor group (young vs old), and the within-subjects factors stimulation (sham and tACS) and time (prestimulation, 0, 60 and 120 min after stimulation) were treated as fixed factors for the single pulse TMS model. Intensity was a within-subject fixed factor in the model for the *I*/*O* curve. The inter-stimulus interval (ISI) was excluded as the within-subject fixed factor in a model for SICI and ICF. Asterisks indicate significant results (*p* < 0.05)

*df* degrees of freedom

**Fig. 3:**
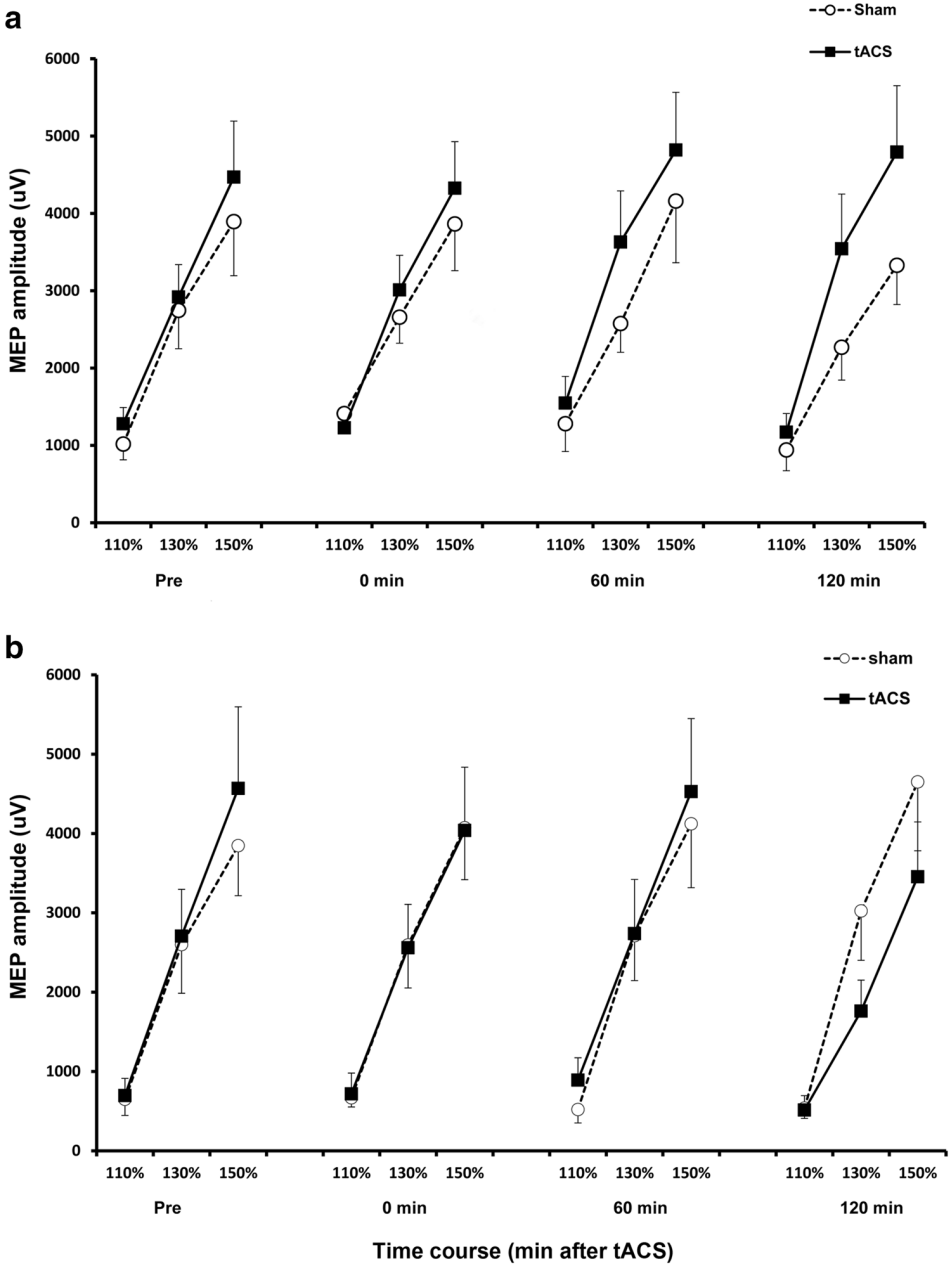
The effect of tACS at iAPF on corticospinal excitability measured by recruitment curves. The *x*-axis displays the RMT (%) for each time point (in min) before and after stimulation (Pre = before stimulation). The *y*-axis displays the MEP amplitude (mean ± SEM as error bars) in µV. **a** Young group: significant effect of tACS stimulation compared to sham 60 min (130% RMT) and 120 min (130 and 150% RMT) after stimulation. **b** Old group: There were no significant changes in the MEP amplitudes between tACS and sham stimulation. Filled symbols indicate MEP amplitudes in the tACS stimulation conditions

In the other measure of corticospinal excitability called the recruitment curve, a monotonous relationship of TMS intensities and MEPs was observed in both groups [significant main effect of intensity: *F* (2, 7344.15) = 2159.34, *p*  ≤ 0.001, *d* = 0.610] (Table 1). This indicated that increasing TMS intensities (in terms of percentage RMT) evoked increasing MEP amplitudes. However, similar intensities did not evoke the same MEP sizes over time as reflected by the changes in the steepness of the recruitment curves [significant main effect of time: *F* (3, 7344.18) = 19.81, *p*  ≤ 0.001, *d* = 0.153, significant time × intensity interactions: *F* (6, 7344.07) = 2.22, *p* = 0.038, *d* = 0.106]. Increase in MEP amplitudes evoked at higher intensities increased the steepness of the curve, while the decrease of these amplitudes decreased the steepness of the curve. The results also showed that the steepness of the recruitment curve was also different between stimulation conditions particularly at later time points [significant main effect of stimulation: *F* (1, 7344.47) = 30.60, *p*  ≤ 0.001, *d* = 0.190 and significant stimulation × time interactions: *F* (3, 7344.21) = 11.28, *p*  ≤ 0.001, *d* = 0.140]. After stimulation, the young group’s recruitment curved became steeper in the tACS condition compared to sham (Fig. 2a), while in the old group the steepness of the recruitment curve was similar except 120 min after stimulation when the steepness of the curve in the tACS condition decreased [significant group × stimulation interactions: *F* (1, 7344.47) = 50.33, *p*  ≤ 0.001, *d* = 0.131 and significant group × stimulation × time interactions: *F* (3, 7344.21) = 26.54, *p*  ≤ 0.001, *d* = 0.093] (Fig. 2b). In the young group, higher intensities evoked bigger MEPs 60 and 120 min after tACS stimulation compared to sham [significant group × intensity interactions: *F* (2, 7344.15) = 23.85, *p*  ≤ 0.001, *d* = 0.305 and significant group × stimulation × intensity interactions: *F* (2, 7344.13) = 16.81, *p*  ≤ 0.001, *d* = 0.048]. The MEP amplitudes significantly increased at intensities equal to 130% RMT [*t* (11) = − 2.937, *p* = 0.014] 60 min after stimulation and 130% RMT [*t* (11) = − 2.558, *p* = 0.027] to 150% RMT [*t* (11) = − 2.614, *p* = 0.024] 120 min after tACS stimulation in the young group [significant four-way interaction of group × stimulation × time × intensity: *F* (6, 7344.07) = 5.16, *p*  ≤ 0.001, *d* = 0.214]. In contrast, there were no significant differences in the recruitment curve before and after stimulation in the old group in both stimulation conditions (Fig. 3).

### Intracortical excitability

**Fig. 4:**
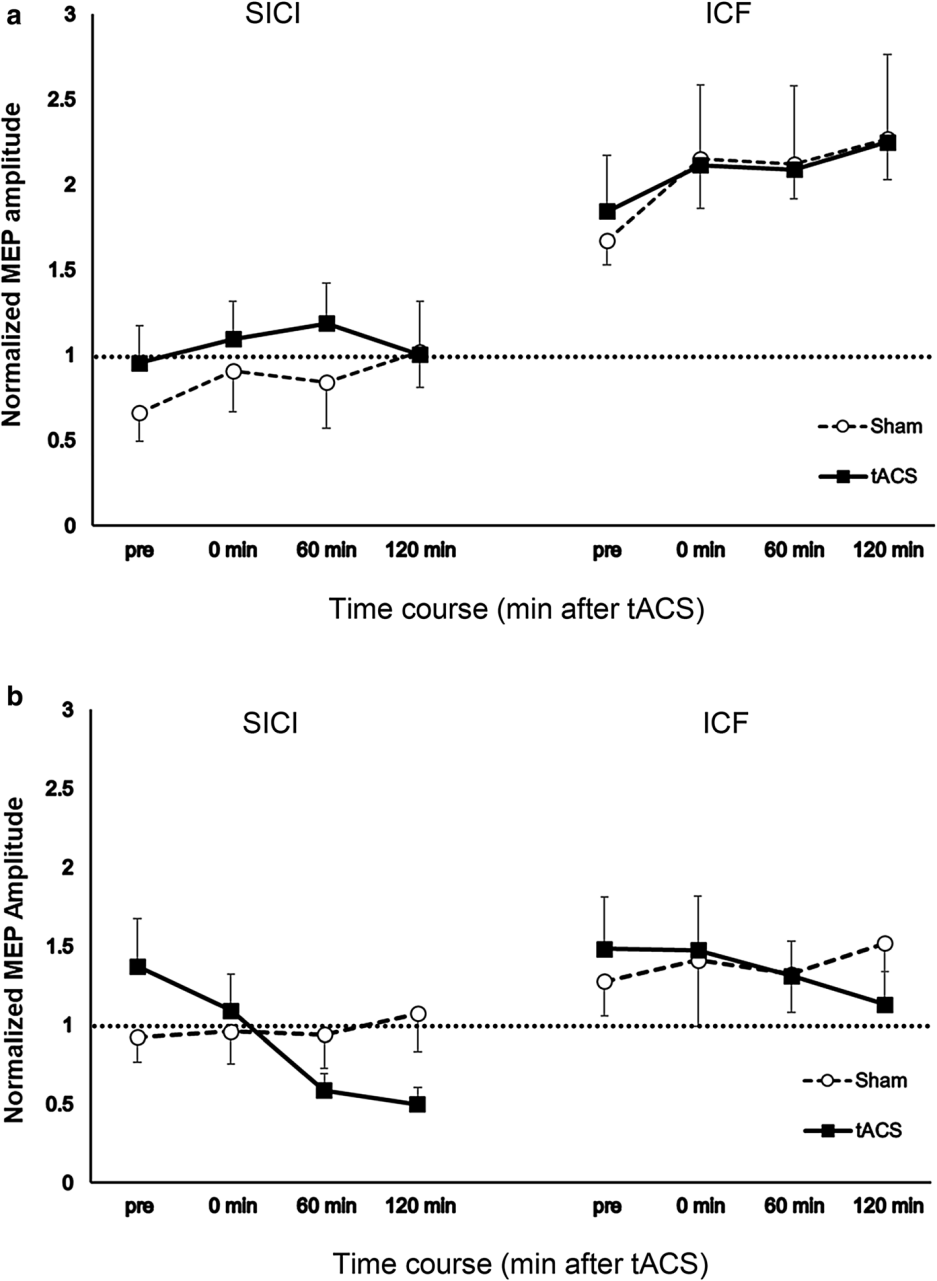
The effect of tACS at iAPF on intracortical excitability. The *x*-axis displays the short interval intracortical inhibition (SICI) on the left and short interval intracortical facilitation (ICF) on the right for each time point (in min) before and after stimulation (Pre = before stimulation). The *y*-axis displays the MEP amplitude (mean ± SEM) normalized to the test pulse (SI 1 mV). **a** Young group: tACS stimulation decreased SICI. **b** Old group: tACS increased SICI post stimulation. Filled symbols = tACS stimulation, open symbols = sham conditions

The results of the linear mixed model analysis showed that changes in MEP amplitudes evoked by paired-pulse TMS with 2 ms ISI were significant over time [significant main effect of time: *F* (3, 2437.26) = 5.04, *p* = 0.002, *d* = 0.164]. As shown in Fig. 4, inhibition was significantly different in magnitude between sham and tACS conditions particularly after stimulation [significant main effect of stimulation: *F* (1, 2437.23) = 7.24, *p* = 0.007, *d* = 0.162 and stimulation and time interactions: *F* (3, 2437.50) = 15.80, *p*  ≤ 0.001, *d* = 0.237]. SICI decreased in the young group, whereas it increased in the old group after tACS stimulation, while no significant changes were observed in SICI after sham stimulation [significant group × stimulation interactions: *F* (1, 2439.46) = 7.92, *p* = 0.005, *d* = 0.259 and significant group × time interactions: *F* (3, 2437.25) = 17.19, *p*  ≤ 0.001, *d* = 0.196]. Specifically, inhibition in the young group decreased 60 min [*t* (2432.281) = 0.304, *p*  ≤ 0.001] after tACS stimulation, while it increased 120 min [*t* (2432.242) = 0.093, *p* = 0.001] after tACS stimulation in the old group compared to inhibition at the same time point after sham stimulation [significant group × stimulation × time interactions: *F* (3, 2437.20) = 6.33, *p*  ≤ 0.001, *d* = 0.678]. There was no significant change in SICI in the sham condition in both groups. For ICF, the analysis showed significant differences in MEP amplitudes between the two groups [significant main effect of group: *F* (1, 23.99) = 6.56, *p* = 0.017, *d* = 0.744]. Overall, both groups exhibited facilitation, however the magnitude was higher in the young group (mean ± SE 2.05 ± 0.21 μV) than the old group (mean ± SE 1.31 ± 0.21 μV) [significant main effect of time: *F* (3, 2511.52) = 5.61, *p* = 0.001, *d* = 0.145 and significant group and time interactions: *F* (3, 2511.51) = 5.60, *p* = 0.001, *d* = 0.278]. The main effect of stimulation was significant [*F* (1, 2512.15) = 11.80, *p* = 0.001, *d* = 0.215] indicating differences in facilitation between sham and tACS condition. However, the difference was not robust (mean ± SE sham − 1.60 ± 0.15 μV; tACS − 1.76 ± 15 µV) and no significant other interactions with the factor stimulation were observed (Table 1).

In summary, tACS stimulation markedly increased corticospinal excitability compared to sham in both groups in the single-pulse TMS paradigm. However, only the young group exhibited increased excitability in the recruitment curved after tACS stimulation. TACS has an age-dependent effect on SICI, as inhibition decreased in the young group but increased in the old group. No significant changes in ICF were observed in both groups after sham and tACS stimulation.

## Discussion

The present study aimed at exploring the impact of tACS stimulation applied at iAPF on motor cortex excitability. We observed an effect of the stimulation on several measures of motor cortex excitability (single-pulse TMS, recruitment curve and SICI/ICF) that differed between young and old participants. In particular, we found a comparable increase in corticospinal excitability after tACS stimulation in both groups (single-pulse TMS and recruitment curve). With regard to intracortical excitability, we observed that tACS stimulation increased SICI in the old group, while decreasing it in the young group. We argue that these effects might be due to an upregulation of the alpha activity due to the stimulation.

### Modulation of motor cortex excitability in young individuals

In the young group, tACS applied to the motor cortex at iAPF for 10 min markedly increased corticospinal excitability (increase in MEP amplitudes) as measured by single-pulse TMS. On the other hand, corticospinal excitability also increased after sham stimulation but the magnitude was less than after tACS and did not reach significance. We would argue that the effect of tACS might have been caused by the up-regulation of the alpha oscillation in the motor cortex due to neuronal entrainment. Previous tACS studies consistently reported long-lasting increase in EEG alpha power for up to 70 min after alpha tACS stimulation of the parieto-occipital areas (Zaehle et al. 2010; Neuling et al. 2012a; Helfrich et al. 2014; Vossen et al. 2015; Kasten et al. 2016). This scenario may apply to the motor cortex as well, because it has a distinct propensity to oscillate in the 7–14 Hz frequency range (Castro-Alamancos 2013). In vitro and in vivo recording in rats have shown that afferent activity between 7 and 14 Hz can trigger augmenting responses in the motor cortex that are maximal during anesthesia, slow-wave sleep and quiet periods of awake immobility but not during an activated state such as movement (Castro-Alamancos and Connors 1996). Based on current source density analysis, current flow in the motor cortex during 7–14 Hz oscillations is strongest in layers II–III and V in animals (Castro-Alamancos and Rigas 2002). In humans, the cortical pyramidal neurons in layers III and V are implicated in the generation of action potentials and I waves during TMS stimulation (Seo et al. 2016). Therefore, we would expect an increase in alpha activity and increased corticospinal excitability after tACS since the impact of stimulation will be strongest on these layers. For instance, a general increase in theta/alpha power density paralleled the increase in excitability in the motor cortex of young participants after the application of slowly oscillating transcranial direct current (so-tDCS) (Pellicciari et al. 2013). Based on these results, we believe that alpha oscillation in the motor cortex was upregulated in our young participants. However, we could not directly show changes in the alpha power after stimulation, since we do not have post-stimulation EEG recordings. We decided against a measurement of post-stimulation EEG data because an influence of the TMS measurement on the EEG cannot be completely ruled out (Stamoulis et al. 2011). EEG data recorded after TMS interventions if available must be carefully interpreted. It should be noted that not all previous studies using tACS in the alpha frequency range found an increase in cortico-spinal excitability (Antal et al. 2008; Zaghi et al. 2010; Wach et al. 2013). TACS over the primary somatosensory cortex using the participant’s mu-alpha frequency also failed to elicit an EEG mean power increase (Gundlach et al. 2017). In rat motor cortex, they have shown that applying the same subthreshold tACS waveform to the same cortical area did not always elicit the same effects on cortical excitability. Cortical excitability rather depended on the parameters of the suprathreshold pulse train used to probe cortical excitability (Khatoun et al. 2017). In our study, we expected stronger effects on neuronal excitability compared to previous studies because we used stronger current intensity and a stimulation frequency tailored to the individual (1.5 mA at iAPF). The latest neural network plasticity model of tACS effects also suggests that stimulation with frequencies near the person’s alpha peak frequency leads to greater effects (Vossen et al. 2015).

In the other measure of corticospinal excitability (recruitment curve), real tACS stimulation also induced an increase in MEP amplitudes compared to sham stimulation in the young group. This result is in line with the findings of previous tDCS/tACS-EEG studies that showed an increase in the steepness of the recruitment curve after stimulation (Neuling et al. 2012a, b; Pahor and Jaušovec 2014). Compared to the excitability measured from a small group of motor cortex neurons at threshold intensity (SI 1 mV), changes in the recruitment curve reflect the activation or recruitment of additional neurons in the vicinity of the motor hotspot (Di Lazzaro et al. 1998). These changes in the recruitment curve of young participants are compatible with the widespread effects of neuronal entrainment. During stimulation, entrainment of alpha oscillations could spread to distant motor cortex neurons via cortico-cortical axonal propagation through the sulci or gyri as demonstrated in simulation and EEG studies in humans (Hindriks et al. 2014). Transcranial sinusoidal oscillating current stimulation in anesthetized and behaving rats also caused a widespread entrainment of cortical areas including the hippocampus (Ozen et al. 2010).

In the measures of intracortical excitability, paired-pulse stimulation with 2 ms ISI (SICI) supressed cortical excitability (smaller MEPs), whereas paired-pulse stimulation with 13 ms ISI (ICF) increased cortical excitability (bigger MEPs) at baseline in the young group. This was an expected finding among healthy young individuals where the balance between the inhibitory and excitatory circuits is assumed to be intact (Kujirai et al. 1993; Ziemann et al. 1996a). However, 60 min after the stimulation tACS caused a significant reduction of inhibition in the young group but no difference in facilitation. Similar results were observed using anodal tDCS, which can increase the excitability of the primary motor cortex (long-term potentiation or LTP-like effect). Here, SICI was reduced while ICF was enhanced (Nitsche et al. 2005). Magnetic resonance spectroscopy (MRS) could show that the excitatory effect of anodal tDCS was due to a local reduction in GABA levels (Stagg et al. 2009). In cortical networks, single GABA neurons are efficient inhibitors of pyramidal cell firing. A net decrease in GABA-mediated inhibition could transiently liberate the pyramidal cell resulting in greater facilitation (Gonzalez-Burgos and Lewis 2008). Additionally, animal studies have shown that reduction of inhibition is a necessary step in the induction of LTP-like plasticity in the horizontal pathways in the motor cortex (Jacobs and Donoghue 1991; Castro-Alamancos et al. 1995; Trepel and Racine 2000; Bachtiar and Stagg 2014). Based on this evidences, we suggest that the change in motor cortex excitability in the young group might be due to the reduction of GABA-mediated inhibition in the motor cortex after tACS stimulation at iAPF. This could also explain the increased excitability in the single-pulse TMS and *I*/*O* curve paradigms.

### Modulation of motor cortex excitability in old individuals

In the old group, tACS at iAPF also increased corticospinal excitability in the single-pulse TMS paradigm and its magnitude was comparable to that of the young group. There was no significant changes observed after sham stimulation. Our findings in the tACS condition was contrary to our expectations. We had assumed that the old group would show a greater increase in excitability than the young group because they should have profited more from the upregulation of their reduced alpha power. This result does not fit well with the observed state-dependent effect of tACS in the parieto-occipital area: stimulation only enhanced EEG alpha power and coherence under conditions of low alpha power (eyes-open) and not under conditions of high alpha power (eyes-closed) (Neuling et al. 2013; Ruhnau et al. 2016). It is possible that the old group’s prestimulation iAPF although low when compared with the young group still represents their optimal resting oscillatory frequency and therefore does not represent a relative state of low alpha activity within their age group. Alternatively, their mu rhythm’s amplitude might be high or the same as the iAPF from the parietal area. In this case, smaller MEPs will be elicited by the magnetic pulse (Sauseng et al. 2009). The increase in MEPs after tACS stimulation (corticospinal excitability increase) was comparable to the young group, although the old group received stimulation at lower frequencies. This suggests that tACS at iAPF may upregulate the elderly participant’s excitability to some degree. Furthermore, the increase in excitability after stimulation also indicates an intact cortico-spinal pathway in the old group. It is possible that our old participants have not yet undergone age-related changes in the corticospinal tract, such as a decrease in synaptic density, a decrease in the motor units firing rates, as well as an altered mode of recruitment and decruitment in the FDI (Eisen et al. 1996; Erim et al. 1999).

Regarding the recruitment curve, the old group exhibited no significant changes after stimulation, whereas there was a significant increase in the steepness of the *I*/*O* curve for the young group. The increased cortical excitability in single-pulse TMS paradigm without an accompanying *I*/*O* curve changes in the old group after stimulation suggests an age-related degradation of pathways within the motor cortex. Different from the single pulse TMS, which depends upon an intact cortico-spinal pathway, the *I*/*O* curve reflects the activation of additional neurons in the vicinity of the motor hotspot. In the human motor cortex, there is a reduction in the number of synapses, the size of compound excitatory post-synaptic potentials (i.e. the number of cortical neurons in the motor cortex that converge on a single corticospinal motoneuron), as well as prolongation of the post-synaptic contact zone from middle age onwards (Adams 1987; Eisen et al. 1996; Todd et al. 2010). These microstructural changes in the motor cortex plus the widespread reductions in gray matter volume, as well as a deterioration in white matter microstructure in the brain (Masliah et al. 1993; Giorgio et al. 2010), could contribute to the impaired recruitment of neurons distant to the site of stimulation in the old group, in spite of an intact corticospinal tract.

With regard to SICI and ICF, our old participants exhibited a reduced SICI but intact ICF at baseline. This finding is in line with some of the previous TMS studies showing reduction of SICI in the elderly (Peinemann et al. 2001; Heise et al. 2013). However, there are also divergent results (Kossev et al. 2002; Wassermann 2002; Oliviero et al. 2006; Smith et al. 2009; McGinley et al. 2010). The discrepancies in the results could be due to differences in the experimental setup [use of monophasic vs biphasic TMS waveforms, different interstimulus intervals (ISI), measurement from the right or left-brain hemisphere] and the different mean age of elderly participants across the studies. Long-interval intracortical inhibition (LICI) and cortical silent period (CSP) which are measures of GABA_B_ receptor-mediated inhibition were found to be increased and shortened with increasing age, respectively (Sale and Semmler 2005; Oliviero et al. 2006; Silbert et al. 2006; McGinley et al. 2010). In the old group, tACS stimulation at iAPF reduced the MEP amplitudes in the SICI paradigm, which indicates increased GABA_A_ receptor-mediated inhibition (Kujirai et al. 1993; Ziemann et al. 1998). This is compatible with the view that our results are due to a modulation of the alpha oscillation, because the alpha oscillation is thought to be generated or regulated by GABA interneuron-mediated synaptic inhibition (Jones et al. 2000; Schreckenberger et al. 2004; Ahveninen et al. 2007; Fanselow et al. 2008; Lőrincz et al. 2009; Lozano-Soldevilla et al. 2014). GABAergic interneurons are also implicated in the generation of gamma oscillations. Driving gamma oscillations using tACS modulates inhibition in the human motor cortex (Nowak et al. 2017). Similarly, studies with rodents have shown that exposure to an extremely low-electromagnetic field (ELEMF) with alternating 10 and 16 Hz frequencies increased and intensified the burst structure in rodent’s cortical in vitro networks, as well as increasing the population of GABAergic neurons (Gramowski-Voss et al. 2015). We also suggest that the net increase in inhibition in the motor cortex of the old group due to the stimulation was responsible for the lower facilitation of MEP amplitudes in the single-pulse TMS paradigm, the MEP recruitment in the *I*/*O* curve and possibly the ICF, which remained unchanged after stimulation in the old group.

## Conclusion

Our study was the first to compare the after-effects of tACS on corticospinal excitability and intracortical inhibition and facilitation in old and young healthy adults. Results showed the effect of tACS applied at iAPF on corticospinal excitability measured by single-pulse TMS is not state-dependent, since young and old participants exhibited a similar increase in excitability after stimulation. On the other hand, the differences between old and young with regard to the recruitment curve might be secondary to age-related degradation of pathways within the motor cortex. The differences with regard to SICI and ICF indicate that the mechanism behind the stimulation effects might be different between the two groups. GABA-mediated inhibition was decreased after stimulation in the young group, whereas it was increased in the old group after stimulation. Capitalizing on these findings, tACS can be considered a promising tool, which can safely modulate oscillatory activity for improving cognition and for treatment purposes. In young adults, the reduction of GABA-mediated inhibition in the sensorimotor cortex was shown to be beneficial for motor sequence learning (Floyer-Lea et al. 2006). In the elderly, parietal alpha tACS selectively and significantly improved performance in a working memory paradigm that probed inhibitory abilities (Borghini et al. 2018). Finally, enhancing intracortical inhibition could depress dysfunctional hyper-excitability in the cortex associated with the increased incidence of epilepsy (Stephen and Brodie 2000; Romei et al. 2008; Thut and Miniussi 2009; Leppik and Birnbaum 2010).

## Electronic supplementary material

Below is the link to the electronic supplementary material.

Supplementary material 1 (PDF 89 KB)

Supplementary material 2 (PDF 133 KB)
