# Supplementary material for: The effects of transcranial alternating current stimulation (tACS) at individual alpha peak frequency (iAPF) on motor cortex excitability in young and elderly adults
Source: Exp Brain Res. 2018 Jun 26;236(10):2573–88. doi: 10.1007/s00221-018-5314-3 (PMC6153871; doi:10.1007/s00221-018-5314-3)
Supplement: Supplementary file 1 — Supplementary material 1 (PDF 89 KB) [file 221_2018_5314_MOESM1_ESM.pdf]

Experimental Brain Research

Supplementary material

The effects of transcranial alternating current stimulation (tACS) at individual alpha peak frequency (iAPF) on motor cortex excitability in young and elderly adults

Shane Fresnoza[1,3]*, Monica Christova[2,4], Theresa Feil[1], Eugen Gallasch[2,3], Christof Körner[1,3], Ulrike Zimmer[1,5], Anja Ischebeck[1,3]

[1] Institute of Psychology, University of Graz, Graz, Austria; [2] Institute of Physiology, Medical University of Graz, Graz, Austria; [3]BioTechMed, Graz, Austria; [4]Institute of Physiotherapy, University of Applied Sciences FH-JOANNEUM, Graz, Austria; [5]Faculty of Human Sciences, Medical School Hamburg (MSH), Hamburg, Germany

1 Akaike Information Criterion (AIC) values

**Single pulse TMS**		**I/O curve**	
Random intercept model only:	82069.061	Random intercept model only:	134551.711
+ Time	81902.664	+ Time	134514.345
+ Stimulation	81530.075	+ Intensity	131264.586
+ Time x stimulation	81438.831	+ Time x Intensity	131262.884
+ Group	**81439.485**	+ Stimulation	131228.403
+ Group x stimulation	81425.688	+ Stimulation x time	131203.832
+ Group x time	81405.003	+ Stimulation x intensity	131198.144
+ Group x stimulation x time	**81409.285**	+ Stimulation x intensity x time	**131205.759**
		+ Group	**131207.245**
		+ Group x time	**131208.895**
		+ Group x intensity	131167.916
		+ Group x stimulation	131122.754
		+ Group x intensity x time	**131131.741**
		+ Group x stimulation x time	131056.276
		+ Group x stimulation x intensity	131026.428
		+ Group x stimulation x time x intensity	131014.705
**SICI**		**ICF**	
Random intercept model only:	5955.649	Random intercept model only:	7947.609
+ Time	5949.870	+ Time	7932.632
+ Stimulation	5943.366	+ Stimulation	7924.767
+ Stimulation x time	5903.721	+ Stimulation x time	**7926.361**
+ Group	**5904.744**	+ Group	7922.534
+ Group x stimulation	5898.638	+ Group x stimulation	7921.139
+ Group x time	5855.125	+ Group x time	7910.525
+ Group x time x stimulation	5842.224	+ Group x time x stimulation	**7914.736**

Table 1 AIC values of single-pulse TMS, I/O curve, SICI and ICF models. AIC values was determined by adding a factor to the model one-at-a-time. A decrease or increase in AIC value (> 2) upon the addition of a factor indicates model fit improvement or worsening, respectively.
