# Supplementary material for: The effects of transcranial alternating current stimulation (tACS) at individual alpha peak frequency (iAPF) on motor cortex excitability in young and elderly adults
Source: Exp Brain Res. 2018 Jun 26;236(10):2573–88. doi: 10.1007/s00221-018-5314-3 (PMC6153871; doi:10.1007/s00221-018-5314-3)
Supplement: Supplementary file 2 — Supplementary material 2 (PDF 133 KB) [file 221_2018_5314_MOESM2_ESM.pdf]

Experimental Brain Research

Supplementary material

The effects of transcranial alternating current stimulation (tACS) at individual alpha peak frequency (iAPF) on motor cortex excitability in young and elderly adults

Shane Fresnoza[1,3 *], Monica Christova[2,4], Theresa Feil[1], Eugen Gallasch[2,3], Christof Körner[1,3], Ulrike Zimmer[1,5], Anja Ischebeck[1,3]

[1] Institute of Psychology, University of Graz, Graz, Austria; [2] Institute of Physiology, Medical University of Graz, Graz, Austria; [3]BioTechMed, Graz, Austria; [4]Institute of Physiotherapy, University of Applied Sciences FH-JOANNEUM, Graz, Austria; [5]Faculty of Human Sciences, Medical School Hamburg (MSH), Hamburg, Germany

2 Akaike weights

Model	Parameters	AIC_i	Δ_i (AIC)	Relative likelihoods	w_i (AIC)
**S1mV**	Random intercept	82069.061	664.058	0.000	0.000
	+ time	81902.664	497.661	0.000	0.000
	+ stimulation	81530.075	125.072	0.000	0.000
	+ time x stimulation	81438.831	33.828	0.000	0.000
	+ group	81439.485	34.482	0.000	0.000
	+ group x stimulation	81425.688	20.685	0.000	0.000
	+ group x time	81405.003	0	1.000	0.894
	+ group x stimulation x time	81409.285	4.282	0.118	0.106
				Sum = 1.118	
**I/O curve**	Random intercept	134551.711	3537.006	0.000	0.000
	+ time	134514.345	3499.640	0.000	0.000
	+ intensity	131264.586	249.881	0.000	0.000
	+ time x intensity	131262.884	248.179	0.000	0.000
	+ stimulation	131228.403	213.698	0.000	0.000
	+ time x stimulation	131203.832	189.127	0.000	0.000
	+ intensity x stimulation	131198.144	183.439	0.000	0.000
	+ intensity x stimulation x time	131205.759	191.054	0.000	0.000
	+ group	131207.245	192.540	0.000	0.000
	+ group x time	131208.895	194.190	0.000	0.000
	+ group x intensity	131167.916	153.211	0.000	0.000
	+ group x stimulation	131122.754	108.049	0.000	0.000
	+ group x intensity x time	131131.741	117.036	0.000	0.000
	+ group x stimulation x time	131056.276	41.571	0.000	0.000
	+ group x stimulation x intensity	131026.428	11.723	0.003	0.003

	+ group x stimulation x intensity x time	131014.705	0	1.000	0.997
				Sum = 1.003	
**SICI**	Random intercept	5955.649	113.425	0.000	0.000
	+ time	5949.870	107.646	0.000	0.000
	+ stimulation	5943.366	101.142	0.000	0.000
	+ time x stimulation	5903.721	61.497	0.000	0.000
	+ group	5904.744	62.52	0.000	0.000
	+ group x stimulation	5898.638	56.414	0.000	0.000
	+ group x time	5855.125	12.901	0.001	0.000
	+ group x stimulation x time	5842.224	0	1.000	0.999
				Sum = 1.001	
**ICF**	Random intercept	7947.609	37.084	0.000	0.000
	+ time	7932.632	22.107	0.000	0.000
	+ stimulation	7924.767	14.242	0.000	0.000
	+ time x stimulation	7926.361	15.836	0.000	0.000
	+ group	7922.534	12.009	0.002	0.002
	+ group x stimulation	7921.139	10.614	0.005	0.004
	+ group x time	7910.525	0	1.000	0.892
	+ group x stimulation x time	7914.736	4.221	0.121	0.107
				Sum = 1.128	

Table 2 Akaike weights of single-pulse TMS, I/O curve, SICI and ICF models. First, we identified the model with the lowest AIC value (AIC_i). Second, we calculated the AIC difference (Δ_i (AIC) by subtracting each AIC to the AIC_i. The AIC difference was then use to calculate the relative likelihood needed for calculating the Akaike weights (w_i (AIC)). The model with the biggest w_i (AIC) is the best model.
